# Financial impacts and community resources utilization of children with feeding difficulties

**DOI:** 10.1186/s12887-022-03566-x

**Published:** 2022-08-25

**Authors:** June Okada, Erin Wilson, John Wong, Man Luo, Lauren Fiechtner, Meg Simione

**Affiliations:** 1 MGH Institute of Health Professions, Charlestown, MA USA; 2 Division of General Pediatrics, MassGeneral Hospital for Children, 125 Nashua St, Suite 860, Boston, MA 02114 USA; 3 Harvard Medical School, Boston, MA USA

**Keywords:** Pediatric feeding disorders, Pediatric feeding difficulties, Social determinants of health, Dysphagia

## Abstract

**Background:**

To examine the extent to which financial impacts and community resources utilization are associated with pediatric feeding difficulties. We hypothesize that children with feeding difficulties will have more financial impacts and community resources utilization than children without feeding difficulties.

**Methods:**

We conducted a secondary analysis of cross-sectional data from the 2017–2018 National Survey of Children’s Health (NSCH) regarding 14,960 children 0–5 years. NSCH utilized random sampling of families across the United States to collect nationally representative data. Outcomes included out-of-pocket costs, caregivers leaving a job due to the child’s health, food insufficiency, receival of food or cash assistance, and receival of special education and/or developmental services. We used a multivariable logistic regression controlling for sociodemographic factors to examine the associations of feeding difficulties with financial impacts and community resources utilization outcomes.

**Results:**

Out of 14,690 respondents, children were a mean (SD) age of 2.53(0.03) years and 1.7% reported feeding difficulties. These children had higher odds of having out-of-pocket costs of ≥$1000 (OR: 3.01; 95% CI: 1.61, 5.62), having a caregiver that left a job due to their child’s health (OR: 3.16; 95% CI: 2.01, 4.98), experiencing food insufficiency (OR: 1.67; 95% CI: 1.03, 2.71), and receiving special education and/or developmental services (OR 3.98; 95% CI: 2.46, 6.45) than children without feeding difficulties.

**Conclusions:**

Children with feeding difficulties are more likely to have financial impacts and community resources utilization than children without feeding difficulties. This information can be used to tailor interventions to improve family-centered care and outcomes for children.

## Background

Feeding difficulties in children are the inability or unwillingness to eat a range of age-appropriate foods and are “associated with medical, nutritional, feeding skill, and/or psychosocial dysfunction” [1]. The impacts of feeding difficulties are far-reaching and can have detrimental effects on growth, nutrition, and development as well as a family’s well-being [1–4]. Because multiple variables including individual, relationship, community, and societal factors can affect a child’s feeding, the Social Ecological Model is a useful guide to examine these potential relationships [5, 6]. Most research to date has focused on the relationships between feeding difficulties and individual factors [1, 3, 7–9], but understanding its association with socio-contextual factors is critical for effective management, resource allocation, and public policy recommendations for children with feeding difficulties. While a small set of investigations have shown that families with children who have feeding difficulties experience financial impacts and need to utilize community resources, the literature in this area is limited and many of the studies have small sample sizes or include qualitative analyses that are not generalizable [10–14].

By examining a large, national sample of children, we can investigate the relationship between socio-contextual factors and feeding difficulties. This understanding can help identify modifiable determinants to address in management approaches and ensure that clinicians are making feasible and realistic recommendations for families. In this study, we examine the extent to which financial impacts and community resources utilization are associated with feeding difficulties in children ages 0–5 years. We hypothesize that children with feeding difficulties (1) have higher out-of-pocket costs; (2) have caregivers who left a job due to the child’s health; (3) experience food insufficiency; (4) receive food or cash assistance; and (5) receive special education and/or developmental services compared to children without feeding difficulties.

## Methods

### Study design and data source

This study is a secondary analysis of cross-sectional data from the combined 2017–2018 National Survey of Children’s Health (NSCH) dataset. NSCH is a de-identified, publicly available dataset sponsored by the U.S. Department of Health and the Human Services and Service Administration, Maternal and Child Health Bureau and was conducted by the U.S. Census Bureau [15]. The NSCH annually reports national and state-level data on the physical and mental health of a child between the ages of 0 and 17, their access to quality care, and on a child’s social context. Caregivers were asked to complete a questionnaire regarding a randomly selected child in their household using either a paper or digital copy. The survey was administered in English and Spanish. There was a total of 52,129 surveys submitted between 2017 and 2018 regarding children under 18 years and 14,737 surveys regarding children 0 to 5 years. The sample in this study was limited to children under 6 because early childhood is a critical period for feeding development [16]. This study was reviewed and found to be exempt by the Massachusetts General Brigham institutional review board.

### Feeding difficulty

The main exposure for this study was the presence of a feeding difficulty, assessed through the question “During the past 12 months, has this child had frequent or chronic feeding difficulty with eating or swallowing because of a health condition?” A positive response was considered confirmation of a feeding difficulty.

### Financial impacts

Outcomes regarding financial impacts included out-of-pocket costs for medical and healthcare, whether caregivers left a job due to the child’s health, and experience of food insufficiency. To measure out-of-pocket costs for medical and healthcare, caregivers were asked “Including co-pays and amounts from Health Savings Accounts and Flexible Spending Accounts, how much money did you pay for this child’s medical, health, dental, and vision care during the past 12 months?” Responses were reported in four categories: “none or less than $250”, “$250–$499”, “$500–$999”, “$1000- $5000”, and “more than $5,000”. Responses were then recategorized into: “$0–$999” or “$1000 or more”. This classification has been used across similar studies and analyses [17–19]. Caregivers who left a job due to their child’s health were identified by a positive response to the question, “During the past 12 months, have you or other family members left a job, taken a leave of absence, or cut down on the hours you work because of this child’s health or health conditions?” To determine food insufficiency, caregivers were asked “Which of these statements best describes your household’s ability to afford the food you need during the past 12 months?” Responses were reported in four categories: “we could always afford to eat good nutritious meals”, “we could always offer enough to eat but not always the kinds of food we should eat”, “sometimes we could not afford enough to eat”, and “often we could not afford enough to eat”. Positive responses to the latter three options were significantly associated with worse health outcomes [20] and thus a dichotomized version of this variable was used in our analysis to differentiate no household food insufficiency and any household food insufficiency [21].

### Community resources utilization

Community resources utilization exposures included receival of food or cash assistance and receival of special education and/or developmental services. To determine receival of food or cash assistance, caregivers were asked “Did this child live in a household that received food or cash assistance?” Responses were reported in three categories: “did not receive food or cash assistance”, “received 1-2 types of assistance”, and “received 3-4 types of assistance”. The latter two options were classified as affirmative responses. Receival of special education and/or developmental services was defined as a positive response to either of these two questions, “Is this child currently receiving special services to meet his or her developmental needs such as speech, occupational, or behavioral therapy?” and “Is this child currently receiving services under a special education or early intervention plan, age 1-17 years?” Children under 1 year were not eligible to answer the question regarding special education but were able to answer the question regarding special services.

### Confounding factors

We selected covariates based on the literature and available data from the NSCH, specifically regarding child and family characteristics that have established linkages with the presence of a feeding difficulty and the socio-contextual factors of interest. Covariates included household income level, presence of special health care needs, and age (years) [3, 9, 22]. Income was defined based on whether the family was considered above or below the federal poverty level. Special health care needs status was determined by the Children with Special Health Care Needs Screener, which was included in the NSCH [23].

### Statistical analyses

We described child, caregiver, and household characteristics, then stratified outcomes by feeding difficulty status. We excluded respondents who did not answer our question of interest to determine the presence of a feeding difficulty (*n* = 47); our final sample size was 14,690 children. We used chi-squared tests to compare categorical variables by the presence of a feeding difficulty. We then assessed the association between feeding difficulties and both financial impacts and community resources utilization using an unadjusted logistic regression and adjusted multivariable logistic regression controlling for covariates. We reported odds ratio (OR), 95% confidence intervals, and *p*-values. A two-sided alpha level of 0.05 was used to determine statistical significance in all analyses. To address the complex survey design of NSCH and produce nationally representative results, we used sampling weights, cluster, and stratum variables from NSCH datasets and conducted all analyses using SAS survey procedures [24, 25]. Statistical analyses were performed using SAS, version 9.4 (SAS Institute Inc., Cary, NC, USA).

## Results

Out of 14,960 children, 1.7% reported having feeding difficulties. Table 1 presents the sample characteristics and bivariate associations by the overall study sample and feeding difficulty status. Children with feeding difficulties were older, mean (SD) age of 2.53 (0.03), than children without feeding difficulties, 1.98 (0.14), (*p* < 0.001). There was no significant difference in the prevalence of feeding difficulties between sexes (*p* = 0.12). In our sample, 52.3% of children were white, 23.9% Latinx, 11.5% Black, and 12.3% Asian, American Indian, Alaska Native, Native Hawaiian, Other Pacific Islander or multi-racial. More children with feeding difficulties (50.5%) had SHCN as compared to children without feeding difficulties (9.6%; *p* < 0.001). Our sample included 23.7% of children with feeding difficulties who were born prematurely and 22.3% who had low birth weight versus 11.4% (*p* = 0.01) and 8.2% (*p* = 0.003) respectively in children without feeding difficulties.

**Table 1:** Socio-contextual factors and child and household characteristics among children aged 0–5 years old

	**Overall**	**Feeding Difficulty**	**No Feeding Difficulty**	***p*-value**
Estimated Number (%)	23,582,568	388,874 (1.7)	23,193,684 (98.3)	
***Child, Parent, and Household Characteristics, Mean (SD) or %***^***a***^				
**Child Characteristics**				
Child’s age	2.52 (0.03)	2.53 (0.03)	1.98 (0.14)	**<0.001**
Child’s sex				0.12
Male	51.2	60.3	51.0	
Female	48.8	39.7	49.0	
Race/Ethnicity				0.58
Hispanic	23.9	27.4	23.8	
White, non-Hispanic	52.3	44.5	52.5	
Black, non-Hispanic	11.5	13.3	11.5	
Other/Multi-racial, non-Hispanics	12.3	14.8	12.2	
Low birth weight	8.4	22.3	8.2	**0.003**
Premature birth	11.6	23.7	11.4	**0.01**
SHCN	10.3	50.5	9.6	**<0.001**
**Family and Household Characteristics**				
0–99% Federal Poverty Level of Household	41.8	43.6	41.8	0.75
Highest Level of Education among Adults				0.25
College degree or higher	53.3	46.3	53.4	
Type of Health Insurance				**0.02**
Public Health Insurance only	32.4	38.7	32.3	
Private Health Insurance only	58.0	46.2	58.2	
Public and private Health Insurance	4.7	11.7	4.6	
Uninsured	4.9	3.3	5.0	
***Socio-Contextual Factors, %***^***a***^				
**Financial Impacts**				
Out-of-pocket costs for medical and health care ≥$1000	12.9	34.6	12.5	**0.002**
Left a job due to child’s health	7.5	34.4	7.1	**<0.001**
Food insufficient	29.2	43.7	28.9	**0.01**
**Community Resources Utilization**				
Received food or cash assistance	38.1	50.9	37.9	**0.03**
Received special education and/or developmental services	8.4	43.7	7.8	**<0.001**

^a^Weighted percentages were used to account for survey design

In the unadjusted and fully adjusted models, we found that children with feeding difficulties had higher odds of financial impacts and community resources utilization than children without feeding difficulties (Table 2). In the multivariable model adjusted for income, presence of SHCN, and age, the associations between feeding difficulty status and socio-contextual outcomes were attenuated when compared to the unadjusted models but the trends were similar. Children with feeding difficulties had higher odds of out-of-pocket costs of ≥$1000 (OR: 3.01; 95% CI: 1.61, 5.62), having a caregiver that left a job due to their child’s health (OR: 3.16; 95% CI: 2.01, 4.98), and experiencing food insufficiency (OR: 1.67; 95% CI: 1.03, 2.71) compared to children without feeding difficulties. When examining community resources utilization, we found children with feeding difficulties had higher odds of participating in special education and/or developmental services (OR 3.98; 95% CI: 2.46, 6.45) as compared to children without feeding difficulties. These findings demonstrate the significant relationship between children with feeding difficulties and various socio-contextual factors. Receiving food or cash assistance was not statistically significant in our final model.

**Table 2:** Logistic regression analysis between outcomes and feeding difficulty

	**Unadjusted Models**	**Fully Adjusted Models**^a^
**Outcomes**	**OR (95% CI)**	
Out-of-Pocket Cost for Medical and Health Care ≥$1000	3.70 (2.16, 6.33)	3.01 (1.61, 5.62)
Left a Job	6.90 (4.38, 10.87)	3.16 (2.01, 4.98)
Food Insufficient	1.91 (1.20, 3.05)	1.67 (1.03, 2.71)
Received Food or Cash Assistance	1.70 (1.06, 2.72)	1.80 (0.96, 3.37)
Received special educational and/or developmental services	9.19 (5.45, 15.49)	3.98 (2.46, 6.45)

Reference group = Children without feeding difficulties. *OR* Odds ratio

^a^Models adjusted for income, age, and special health care needs

## Discussion

Our sample identified 1.7% of children ages 0–5 years with feeding difficulties. We found that children with feeding difficulties were more likely to have out-of-pocket costs for medical and health care ≥$1000, caregivers that left a job due to their child’s health, experience food insufficiency, and participate in special education and/or developmental services compared to children without feeding difficulties. These findings highlight the financial impacts and community resources utilization that affect young children with feeding difficulties. By understanding these relationships, we can tailor interventions to address socio-contextual factors to provide family-centered care and improve outcomes for patients and caregivers.

The study’s findings provide further support regarding the association between financial impacts and children with feeding difficulties [2, 10, 12, 14, 22]. This relationship has been similarly identified in a sample of insured families with children who have feeding difficulties [22]. Though this report did not compare children with and without feeding difficulties or include children without insurance, it demonstrated patterns of increased out-of-pocket costs related to uncovered or underinsured medical expenses, supplies such as specialized food and equipment, childcare/treatment services, travel (e.g. gas, tolls, parking), and lost productivity [14, 22]. Families reported spending an average of $125,645 across multiple years caring for their child with feeding difficulties despite having health insurance and experiencing high deductibles for feeding appointments [14, 22]. Extensive additional expenses has also been reported in families with children with SHCN [19]. Previous qualitative studies have highlighted the large proportion of caregivers of children with feeding difficulties that have had to quit work, decline a promotion, cut back on work hours to care for their child [10, 12, 14, 22]. Families describe the inability to sustain full time employment due to the challenges of balancing the needs of their child with work responsibilities as a result of their child’s feeding difficulties [10, 12, 14, 22]. In addition, children with disabilities, including children with autism spectrum disorder (population with high prevalence of feeding difficulties [26]) were more likely to be food insecure than households with children without disabilities. Additional costs of care, disparate access to needed services, and reduced income from job loss are likely contributing to food insecurity in this population [27]. Children who experience food insufficiency have limited or uncertain ability to acquire acceptable foods [28] suggesting that these children may not always have access to various textures and flavors to support developmentally appropriate feeding skills. Similarly, children with food insufficiency have limited or uncertain availability of nutritionally adequate and safe foods [28] which may reduce positive food experiences, further exacerbating underlying feeding difficulties. We postulate that food insecurity in children with feeding difficulties are related to financial burden and challenges accessing foods which can result in direct and indirect negative impacts to feeding development. The financial hardships experienced by families may also continue to play a role in the maintenance of feeding difficulties and underscore the importance of identifying these factors and addressing in treatment.

In adjusted models, we found no association between children with feeding difficulties and receiving food or cash assistance. This lack of association is likely because the qualifying factors for many assistance programs include specifications for income and presence of disability in the household [29]. However, the finding is notable given the persistent relationship between feeding difficulties and experiencing food insufficiency. Children with feeding difficulties who experience food insufficiency may not be receiving food or cash assistance despite needing access to these services. Prior research found that families raising children with disabilities experience food insecurity at higher rates in comparison to families raising children without disabilities [21]. The same study identified higher Supplemental Nutrition Assistance Program (SNAP) participation in families raising children with disabilities yet noted that participation was relatively low with only 55% of income-eligible families receiving SNAP [21]. The Greater Boston Food Bank recently found that approximately half of adults experiencing food insecurity were not using SNAP or food pantries during the COVID-19 pandemic [30]. Although rates of food insecurity and use of such assistances significantly increased with the pandemic, this study highlighted the reality that not all individuals with food insecurity may access support services. Commonly reported barriers to using assistive programs included desire for self-reliance, lack of knowledge or worry regarding paperwork, stigma, and concern over ineligibility [30]. Often families with children with feeding difficulties are required to purchase specialized foods and equipment for feeding. Without supplemental assistance to offset other expenses, treatment recommendations are unfeasible, unrealistic, or have the potential to cause increased financial burden in the population it is meant to serve.

In our study, we found that children with feeding difficulties were more likely to receive special education and/or developmental services. Feeding difficulties are unlikely to resolve without intervention and early identification and treatment can prevent the exacerbation of problems and stop the development of secondary complications [31, 32]. Previous studies have shown that children with developmental delays were significantly more likely to receive Early Intervention than children without developmental delays [33], a service than can manage and treat feeding difficulties. This study further emphasizes the community resources utilization by children with feeding difficulties and underscores the need to ensure screening for feeding difficulties for children who are receiving specialized services. Alternatively, for children who are not receiving specialized services, clinicians need to determine if additional supports are needed to address unmet social needs [34].

Our study presents with several limitations. First, this study was a secondary analysis of cross-sectional data, and we cannot make conclusions on longitudinal associations between socio-contextual factors and the presence of a feeding difficulty. Second, our categorization of a feeding difficulty as well as of financial impacts and community resources utilization were limited by the responses provided in the survey. Because this was based on caregiver report, we were not able to determine if children had a formal diagnosis of a pediatric feeding disorder thus were restricted to the term “feeding difficulties”. A recent study based on a population of insured families in two states predicted the annual prevalence of pediatric feeding disorders was approximately between 2.7–4.3% [35]. Our study estimated 1.7% of children in the United States present with feeding difficulties, which is lower than what was previously published, and our study may be underreporting children with feeding difficulties.

## Conclusions

Our quantitative analysis of data from a large national sample demonstrates that children ages 0–5 years with feeding difficulties have greater financial impacts and community resources utilization than children without feeding difficulties. More specifically, children with feeding difficulties have out-of-pocket costs more than $1000, have caregivers who left a job due to their child’s health, experience food insufficiency, and receive special education and/or developmental services as compared to children without feeding difficulties. To our knowledge, this study is one of the first analyses to utilize a nationwide dataset to evaluate the relationship between socio-contextual factors and children with feeding difficulties. The findings identify specific factors that clinicians may target during treatment to address children and family’s unmet social needs that impact the child’s feeding. By understanding this information, we can better provide holistic, tailored treatment approaches that account for socio-contextual factors associated with feeding difficulties as early as possible and reduce lasting consequences.

## Data Availability

The datasets analyzed during the current study are available from the Data Resource Center for Child & Adolescent Health, https://www.childhealthdata.org. The Data Resource Center for Child & Adolescent Health requires permission, and this was obtained prior to conducting analyses.

## Abbreviations

NSCH: National Survey of Children’s Health
OR: Odds ratio
SHCN: Special health care needs
SNAP: Supplemental Nutrition Assistance Program
